# Pump-Free Insulin Delivery via an SLA-Printed Hollow Microneedle Patch with an Integrated Self-Sealing Reservoir

**DOI:** 10.3390/mi16121322

**Published:** 2025-11-26

**Authors:** Evie Smith, Naser A. Alsaleh, Mahmoud Ahmadein, Abdullah A. Elfar, Hany Hassanin, Khamis Essa

**Affiliations:** 1 Department of Mechanical Engineering, University of Birmingham, Edgbaston, Birmingham B15 2TT, UKk.e.a.essa@bham.ac.uk (K.E.); 2 Industrial Engineering Department, Imam Mohammad Ibn Saud Islamic University (IMSIU), Riyadh 11432, Saudi Arabia; 3 Department of Production Engineering and Mechanical Design, Tanta University, Tanta 31111, Egypt; 4 School of Science, Psychology, Arts and Humanities, Computing, Engineering and Sport, Canterbury Christ Church University, Canterbury CT1 1QU, UK

**Keywords:** hollow microneedles, pump-free insulin delivery, stereolithography (SLA), 3D printing, self-sealing reservoir, transdermal drug delivery

## Abstract

Hollow microneedle (HMN) systems can deliver insulin with minimal pain, but most rely on external pumps that add bulk, cost, and failure modes. This paper reports the design, fabrication, and mechanical characterisation of a pump-free, refillable HMN patch that integrates a syringe-loadable, self-sealing reservoir and delivers by passive diffusion. A 3 × 4 array of side-orifice conical HMNs with a target height of 1 mm and a bore of 0.8 mm was stereolithography-printed in dental-grade resin and coupled to an elastic-grade resin septum that maintains a leak-free seal after repeated needle puncture. A surface-response design of experiments (DoE) probed wall thickness of 0.10–0.20 mm, post-cure time of 20–60 min, and temperatures of 35–80 °C. The microneedle characteristics include geometric fidelity, insertion into multilayer Parafilm, and axial compression to 150 N. All patches were printed with a hollow channel and side orifices with tips were slightly blunted. Relative to the original design, height undershoot was from −24.5% to −60.5% while base diameters were within −11% to +20%. Parafilm insertion exhibited a peak then force drop at about 0.22 mm displacement with 1.2–1.5 N pierced the first layer. It was found that about 90% of needles penetrated about 381 µm and more than 20% reached 635 µm. Patches withstood 150 N without fracture with strains of 9.7–15.6% and modulus of 8–48 MPa. ANOVA identified wall thickness as a significant factor, with curing temperature not being significant. Contour analysis defined an operating window near a 0.15 mm wall and about 40 min post-cure balancing dimensional fidelity and post-compression height retention. These results define a manufacturable path to compact, pump-free insulin patches with low insertion force and robust mechanics, opening a clinically scalable route to simpler everyday insulin therapy.

## 1. Introduction

Diabetes prevalence and treatment needs continue to rise globally, placing sustained pressure on self-management and health systems [1]. The International Diabetes Federation estimates 589 million adults were living with diabetes in 2024, projecting 853 million by 2050, which underscores the scale of long-term injectable therapy [2,3]. Despite this progress, both modalities impose day-to-day burdens that can erode adherence and glycaemic stability. Needle-related distress is a persistent barrier to initiating and sustaining injection therapy, with many adults reporting fear, anticipated pain, or anxiety that translates into delayed starts or missed doses [4,5]. Lipohypertrophy at injection sites is common and clinically consequential, altering pharmacokinetics and amplifying glycaemic variability and hypoglycaemia risk when insulin is delivered into affected tissue [6]. Evidence indicates substantial prevalence and identifies preventable risks such as poor site rotation and needle reuse, linking everyday workflow and education to safety and efficacy [7].

Hollow microneedles offer a middle ground between hypodermic injection and passive patches by piercing the stratum corneum and delivering liquid into viable skin [8]. Microneedle insertion was found to be substantially less painful than conventional needles, with needle length strongly influencing pain scores [9]. In addition, microfabricated microneedles can be pressed into the skin with minimal or no pain, which establishes a user-acceptance foundation for frequent-use therapies [10].

Clinical feasibility for insulin is supported by proof-of-concept human studies in type 1 diabetes where hollow microneedles delivered rapid-acting insulin and lowered postprandial glucose effectively [11]. In adults with type 2 diabetes, intradermal insulin via a microneedle device improved pharmacokinetics versus subcutaneous injection, suggesting faster onset and better post-meal control [12]. Many hollow microneedle platforms still rely on auxiliary actuation such as springs, syringes, or on-body pumps to drive flow, which adds bulk, complexity, and cost to a skin interface that could be simpler [13]. Active variants also pair microneedles with iontophoresis to push charged drugs, which shows the dependence on external energy for consistent dosing [14]. Tip bores are vulnerable to clogging by corneocytes or compressed tissue during insertion. Side-opening architectures were introduced to mitigate tip occlusion while preserving structural strength and have been adopted in newer designs to minimise clogging risk [15]. Canonical silicon implementations of out-of-plane hollow microneedles demonstrated robust liquid transport and informed later polymer and additive approaches [16].

Insertion penetration force, depth, and failure modes depend on length, wall thickness, and tip radius, so optimisation is essential to ensure reliable intradermal placement without fracture or buckling [17]. Real-time imaging shows that during microneedle injections, the skin behaves as a deformable porous medium, with local expansion absorbing fluid rather than rupturing [18]. Stereolithography enables rapid iteration of microneedle geometries with sub-millimetre accuracy at low tooling costs [19]. Microfabrication demonstrates high-fidelity small-scale features and process control that inform tip fidelity and surface quality but is limited to 2D geometries [20,21,22,23,24]. Materials for printed microneedles currently include biocompatible photopolymers with moduli suitable for rigid hollow needles [25].

Vaccine packaging shows that commercial vial stoppers maintain self-sealing and acceptable fragmentation rates after many punctures, which supports the feasibility of repeated refills in compact devices [26]. Microneedle patches with integrated microfluidics have demonstrated painless transdermal dosing without bulky syringes during use, which reduces the actuation burden for diabetes management [27]. Side-orifice architecture is central to maintaining patency during insertion because an off-apex outlet reduces the chance that the aperture is sealed by a compressed stratum corneum [28]. Rational design of hollow tips corroborates that modified geometries near the apex mitigate skin-induced clogging and stabilise flow at matched lumen sizes [29]. Recent micro- and nano-printed needles with multiple side openings report the elimination of complete clogging events and reduced delivered-volume variability [30,31]. Comparative work indicates that Parafilm M and Strat-M provide practical simulants for methodical iteration before vivo testing, enabling consistent layer-by-layer penetration mapping and force–displacement profiling. Optimisation studies show that print orientation and post-processing sharpen tips and improve dimensional fidelity in moulds and arrays [32]. Directly printed hollow arrays demonstrate sufficient axial strength, porcine-skin penetration, and efficient liquid delivery, indicating that rigid photopolymer needles can achieve both mechanical robustness and hydraulic performance [33].

Translation remains constrained by systems that depend on external actuation or active dispensing, which increases on-body complexity and introduces additional failure modes [34,35,36]]. Contemporary reviews of printed hollow microneedles emphasise opportunities for compact and customizable devices [37]. In addition, studies continue to explore auxiliary energization such as oscillation to stabilise penetration and flow, which signals reliance on actuation rather than purely passive delivery [38]. To bridge these gaps, this study formulates four research questions. What process window yields arrays with open lumens and side orifices and minimal height error relative to the model using SLA materials and cure conditions? What insertion forces and layer-by-layer penetration fractions are achieved across a standardised surrogate within the depth range targeted for intradermal insulin delivery? How much axial strain accumulates at high loads before fracture, and how much height is recovered after unloading to ensure on-body reliability during everyday handling? How do wall thickness, post-cure time, and temperature rank in their influence on geometry, insertion, and mechanical properties, and what combination defines a practical operating point for translation? The aim of this research is to deliver a compact refillable hollow microneedle patch that operates without an external pump, together with a design of an experiments-driven map from process parameters to functional performance and an optimised window for manufacturability.

## 2. Methodology

The methodology proceeded through device design and print preparation on a stereolithography (SLA) desktop printer; fabrication across a design of experiments and visual inspection against predefined rejection criteria, metrology, surface conditions, and outlet patency; insertion testing on skin-like film and axial compression; and data processing with statistical analysis.

### 2.1. Device Design and Materials

**Figure 1:**
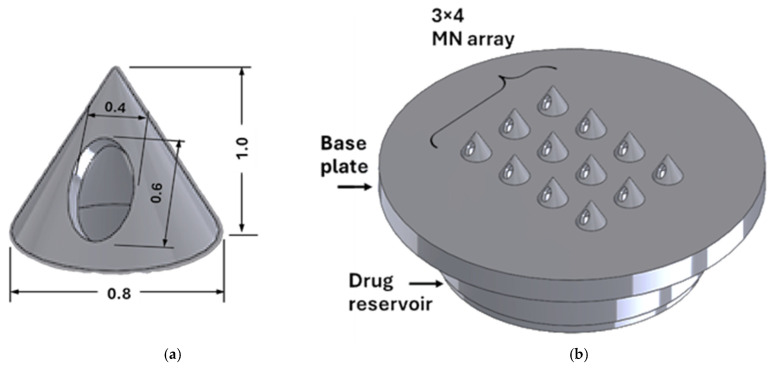
CAD model of (**a**) an individual MN and (**b**) the MN array.

The patch integrates a circular reservoir, a compact manifold, and a 3 by 4 hollow microneedle array designed for refillable and pump-free insulin delivery. Each microneedle is a tapered cone with a single side orifice positioned close to the tip, so the sharp apex opens tissue while the outlet remains patent during entry; see Figure 1. This geometry supports passive liquid diffusion and reduces the risk of skin-induced clogging during insertion. Target dimensions are fixed, so downstream experiments isolate the effect of wall thickness. The side orifice is an oval of 0.4 by 0.6 mm on the distal shaft to balance flow and strength. The patch outer diameter is 15 mm to match a universal microneedle applicator footprint. In total, fifteen nominally identical patches were fabricated, each consisting of a 3 × 4 array of 12 hollow microneedles.

The above constants are summarised concisely in Table 1, together with their functional justifications. Geometric targets are fixed to isolate the effect of wall thickness in subsequent experiments. Needle height is 1 mm, with a bore base diameter of 0.8 mm. Array spacing follows a pitch-to-radius ratio of 3.5 to mitigate the bed of nails effect while maintaining manifold permeability. Reservoir and interface choices capture the trade-off between compactness, refill ability, and sealing reliability. The reservoir outer diameter is 11 mm within the 15 mm patch to preserve an adhesive margin. The calculated reservoir volume is 117.81 mm^3^, which corresponds to 11.81 insulin units when 1 unit equals 0.01 mL. Chamfers at each needle inlet reduce pressure losses and support resin drainage during post-processing. Refilling uses a press-fit elastomeric septum in Elastic 50A with a disc thickness of 1.5 mm and a diameter of 8 mm.

Materials are selected for role-specific performance. Dental SG (Formlabs Inc., Somerville, MA, USA) is used for microneedles and the patch body to deliver a robust axial response and lumen fidelity in hollow features. Elastic 50A (Formlabs Inc., Somerville, MA, USA) is used for the resealable lid to provide repeatable self-sealing during syringe access. Parafilm M (Agar Scientific Ltd., Rotherham, UK) is the insertion surrogate for functional testing. Isopropyl alcohol (IPA; ReAgent Chemical Services Ltd., Runcorn, UK) is the wash solvent in post-print processing. Adhesive tape (Agar Scientific Ltd., Rotherham, UK) secures the base plate to test substrates where required. Design simplification was evidence-led. The final array therefore uses conical needles with a through hole and a single side orifice. Minimum printable wall thickness for patent lumens is 0.10 mm. This study carries three wall levels, which are 0.10 mm, 0.15 mm, and 0.20 mm, while height, bore base diameter, array size, and reservoir diameter remain fixed.

### 2.2. Stereolithography Additive Manufacturing and Post-Processing

**Figure 2:**
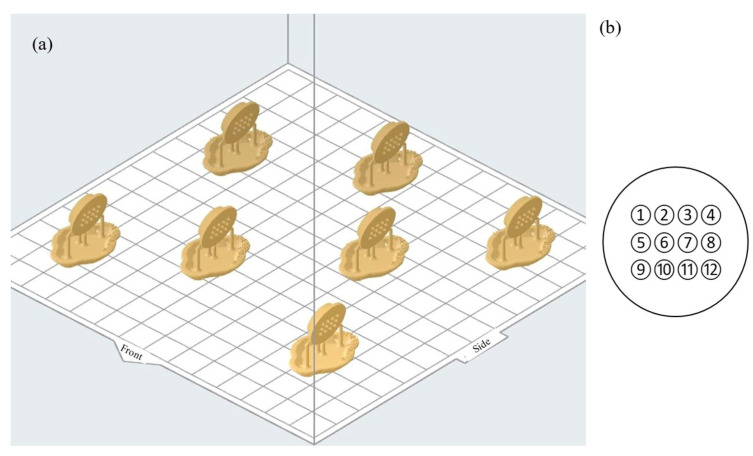
(**a**) SLA slicing setup with 0.2 mm wall thickness. (**b**) Microneedle numbering system.

Stereolithography using Formlab 2 used Dental SG with build preparation defined in PreForm. Printing parameters were fixed after six iteration cycles that explored build angle and layer thickness together with minor design adjustments. Printing direction had a strong effect on needle formation, which is consistent with resin flow over tips during recoating. A 15-degree build angle produced the most uniform formation across the 3 by 4 array and was locked for all experimental builds. The complete PreForm scene for the locked configuration is shown in Figure 2a. Layer thickness was set to 50 µm to maximise tip fidelity and to reduce stair stepping at the side-orifice edge. Supports were minimised to reduce post-processing risk. Four supports per array were used with small touchpoints placed at the patch perimeter so removal forces did not act across needles or near the orifice. This placement preserved lumen patency and protected tips during handling. The support layout, tilt, and slicing settings are shown in Figure 2a.

Print preparation was locked before the experimental matrix. The locked setup includes the 15-degree orientation, the 50 µm layer thickness, the peripheral support scheme, and a standardised wash-and-cure sequence. Locking ensured that the controlled factors in the design of experiments were the primary sources of variation and that outcomes reflected geometry and post-cure conditions rather than setup drift. After printing, parts were washed in isopropyl alcohol in a Form Wash for 20 min and then allowed to drain so solvent cleared from the hollow lumens. Post-cure used a Form Cure unit. Post-cure time and temperature were treated as process factors within the experimental window of 20 to 60 min and 35 to 80 degrees Celsius. The window spans technical guidance and a published condition and enabled estimation of the effects of time and temperature on mechanical and insertion responses.

Orientation and drainage were managed deliberately to preserve lumen patency. The 15-degree tilt promoted resin runoff along the lumen axis and away from the side orifice. Supports were kept clear of outlet regions and of base inlets. Immediately after washing and again after curing, each array was inspected under the same optical setup used for geometric characterisation to confirm open bores, intact tips, and clean orifice edges. Arrays with tip blunting, blocked lumens, or support scarring across a flow path were rejected before testing. Traceability linked manufacturing and metrology. Each build used a consistent file-naming convention in PreForm that matched the identifiers in the data sheets and the design of experiments run order. The needle indexing grid used for sampling is shown in Figure 2b. Height measurements were taken at needles 1, 2, 5, 8, 11, and 12 to avoid sight-line occlusion. Base diameters were sampled across the grid with selections recorded in the data sheet. Figure 2b provides a one-to-one mapping between the printed array and the measurement plan used downstream. Each patch comprises a 3 × 4 array (twelve microneedles); six microneedles were pre-selected at positions 1, 2, 5, 8, 11, and 12 to span the corners and centre of the array and to avoid occlusion during optical measurement.

### 2.3. Characterisation and Functional Testing

Geometrical characterisation used a Keyence VHX-7000 digital microscope (Keyence (UK) Ltd., Milton Keynes, UK). Arrays were positioned on a flat stage and aligned to the optical axis. The predefined needle index grid from 1 to 12 in Figure 2b governed all sampling to ensure traceability across builds. Heights were measured at needles 1, 2, 5, 8, 11, and 12 to maintain clear sight lines across the array. Height was defined as the axial distance from the patch reference plane to the needle apex along the central meridian. The base diameter was measured from front-view images at matched magnification using a consistent edge-to-edge criterion. Each image carried the build identifier and timestamp, so every measurement mapped one-to-one to the corresponding array. Calibration and microscope settings were held constant for all builds. Surface profilometry verified lumen and side-orifice patency and provided roughness metrics for later analysis. Measurements were taken on an Alicona InfiniteFocus G5 in vertical scanning mode. A top-view scan established the patch reference plane, followed by higher-resolution scans over selected needles. Height maps captured the cone flank and the region spanning each side orifice. Roughness Ra was computed along a defined trace that crossed the cone flank near the orifice and then crossed the patch surface, using the instrument’s standard filtering and evaluation length. Scan locations and evaluation paths were recorded per build to preserve repeatability. This measurement plan was applied to all 15 builds in the response-surface experiment, resulting in a total of 90 individual microneedle measurements (6 per array × 15 arrays).

Insertion testing employed Parafilm M as a standardised surrogate for skin. A strip of Parafilm was folded 8 times to produce a multilayer stack near 1 mm of total thickness with individual layers of 127 µm. The stack was fixed on a rigid platen, and the patch was centred beneath the indenter of an Instron 34TM-30 (Instron Ltd., High Wycombe, UK). The crosshead advanced at 8 mm/s to a peak force of 12 N, then held for 10 s. Force and displacement were recorded synchronously. After each test, the Parafilm stack was unfolded and each layer was imaged to count perforations. Penetration at each layer was computed as perforations divided by 12 needles and expressed as a percentage. The minimum success criterion was 20 percent penetration, consistent with the surrogate standard. All raw images and computed penetration tables were logged against building identifiers. Insertion tests were performed on all 15 builds from the response-surface experiment, with one Parafilm stack per patch, giving *n* = 15 penetration profiles in total.

**Figure 3:**
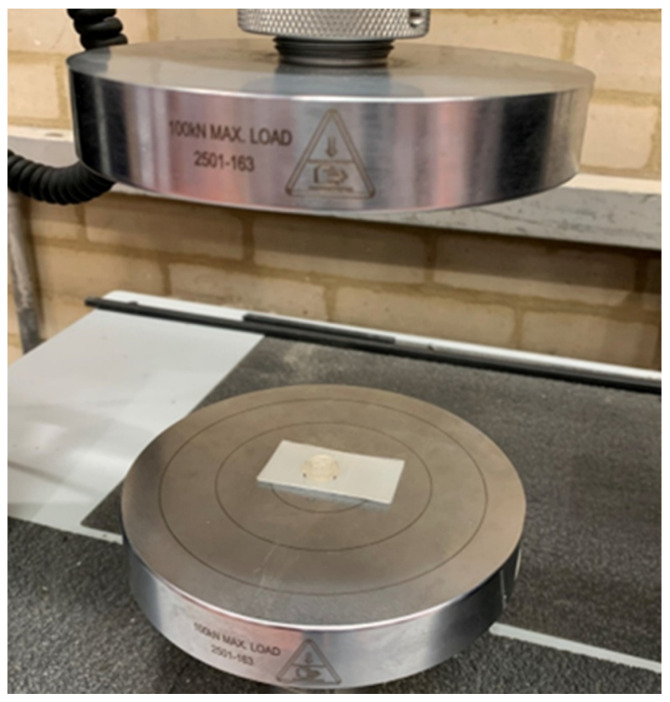
Penetration testing setup with the microneedle patch centred on the lower platen and a multilayer film surrogate positioned between platens for layer-by-layer perforation assessment.

Compression testing characterised the axial response of the arrays independent of insertion. Tests used the same Instron platform with a rigid, polished platen; see Figure 3. The patch was centred and aligned so the load acted normal to the patch plane. Crosshead speed was 0.05 mm per second. Maximum load was 150 N unless a prior failure mode was observed. Stress and strain were derived from measured force and crosshead displacement using the patch footprint as the nominal area and the needle height as the nominal gauge length. The apparent Young’s modulus was calculated from the linear region of the stress–strain response. Compressive strain at maximum load was recorded for each build. Post-test inspections documented any failure initiation sites, such as reservoir, base plate, or needle deformation. Arrays with damage or incomplete tests were flagged and excluded from downstream analysis per the predefined rejection policy.

Quality control gates bracketed the workflow. Pre-test gates required open lumens, intact tips, clean side-orifice edges, and the absence of support scarring across flow paths. Post-test gates required successful data capture, intact fixtures, and valid instrument logs. Any array failing a gate was rejected, and the rejection reason was recorded. The needle index grid in Figure 2b was used consistently for all metrology, so measurements remained directly comparable across wall-thickness groups and post-cure conditions.

### 2.4. Surface-Response Design of Experiments

Design of experiments followed a Box–Behnken response surface plan in Minitab (version 21) to quantify the influence of geometry and post-cure conditions while keeping print count efficient and factor settings within practical bounds. The build setup was fixed so that only the planned variables changed across prints. Geometry varied through wall thickness at three manufacturable and lumen-patent levels. Post-cure time and post-cure temperature were varied within the curing unit’s usable window and within the material guidance range. A total of 15 patches were fabricated to span these conditions under the locked preparation defined earlier. Table 2 defines the response-surface domain. It lists each experimental factor with its symbol, the levels in engineering units, and the rationale for those bounds.

The experimental region respected the spacing strategy, the pitch-to-radius ratio was fixed at 3.5, and pitch values were tied to wall thickness, so spacing scaled coherently with geometry. All other print parameters, wash-and-cure equipment, and metrology settings remained fixed. Each build carried a unique identifier that was propagated to image files, profilometry logs, and the analysis worksheet to preserve one-to-one traceability from raw measurements to design points. Model fitting used a response-surface formulation in Minitab. Model adequacy and assumptions were checked using normal probability plots of residuals, residuals versus fits to assess constant variance, and residuals versus run order to verify independence.

## 3. Results

### 3.1. Geometric Fidelity and Orifice Patency

**Figure 4:**
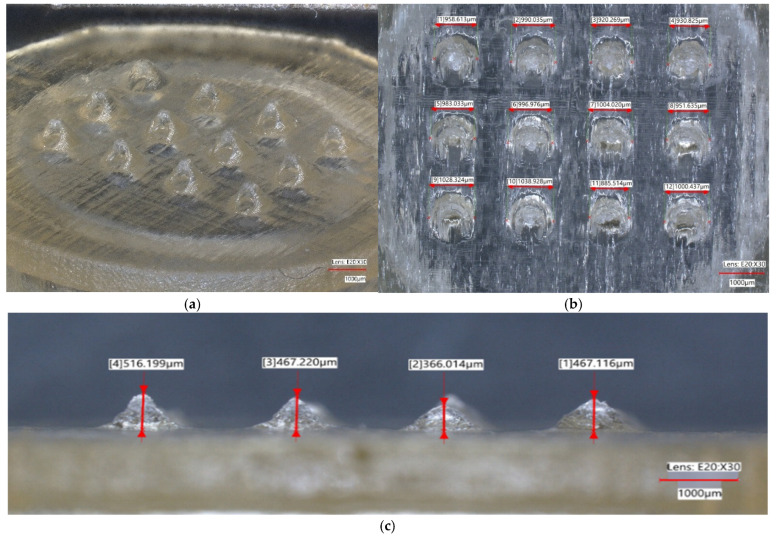
Optical characterisation. (**a**) A 3 × 4 array with uniform cones and slight tip blunting. (**b**) Per-needle base diameters (measured in µm) overlaid on the front view; the values cluster around the 0.8 mm CAD target with deviations of −11% to +20%. (**c**) Per-needle microneedle heights (µm) from the side view. [ ] are the microarray numbers.

Figure 4 presents the optical characterisation of the printed arrays. The oblique view in Figure 4a shows a uniform 3 × 4 array with slight tip blunting and no gross defects at the array scale. The front view in Figure 4b lists per-needle base diameters as on-image overlays in µm. These values cluster around the 0.8 mm CAD target with deviations of −11% to +20% across the arrays shown. For a CAD target of 0.8 mm, this deviation range corresponds to printed base diameters of approximately 0.71–0.96 mm (710–960 µm), which can appear visually close to 1.0 mm in the scaled micrographs even though the numerical overlays report values around 800 µm. The side view in Figure 4c lists cone heights as on-image overlays along a transverse line of needles. Relative to the 1 mm target, the printed heights undershoot by −24.5% to −60.5%. The largest height losses occur at 0.10 mm walls, while 0.20 mm walls show a smaller average shortfall. Across repeated scans, the average bore base diameter is 1.2–16.6% greater than 0.8 mm. These panels together show that broad basal features reproduce more faithfully than the tip region and that height loss is concentrated near the apex rather than at the base.

**Figure 5:**
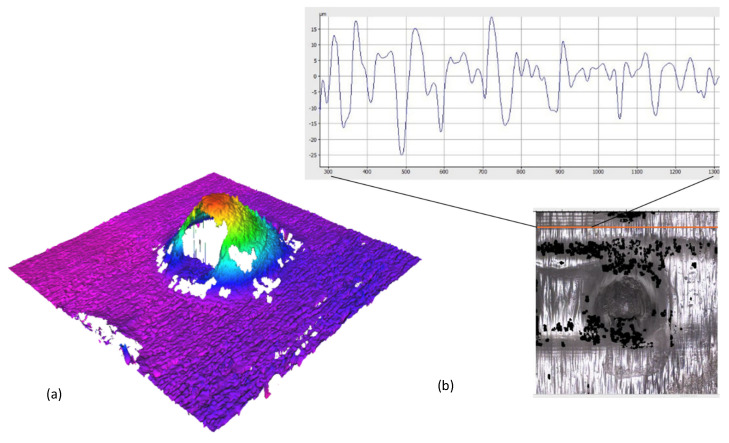
Profilometry. (**a**) Three-dimensional map range from −200 to 1100 µm showing a continuous apex and open outlets. (**b**) Ra 1.8–10.0 µm along the scan line confirms no obstruction. The orange line is the scan line.

Figure 5 provides profilometric confirmation of lumen and side-orifice patency and reports surface conditions. The 3D height map in Figure 5a spans −200 to 1100 µm and shows a continuous apex and smooth transitions from cone to base. No elevated rim or smear is visible over the expected outlet pathway at the instrument resolution. The roughness trace in Figure 5b reports Ra along the indicated scan path with values of 1.8–10.0 µm and does not show an anomalous peak at the apex that would indicate cured residue across the lumen outlet or the side orifice. Both measurements were repeated four times for each wall-thickness group and consistently support patent flow paths after wash and cure. As shown, arrays are uniform at the patch scale. Base diameters remain close to the CAD target with modest oversize on average, while cone heights show systematic undershoot that is most pronounced for thinner walls. Surface topography and roughness measurements corroborate that both the central lumen and the side orifice are open at the resolutions tested.

### 3.2. Insertion Mechanics and Penetration in Parafilm

**Figure 6:**
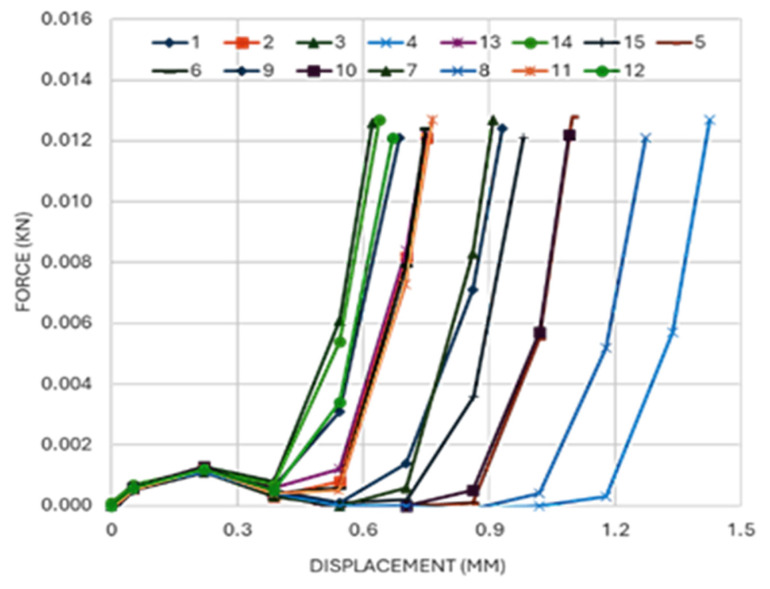
Force–displacement during Parafilm insertion.

Figure 6 shows the force–displacement response during Parafilm M insertion for patches of different wall thicknesses. Curves share a common start with a steady force rise to about 0.2 mm displacement that reflects film deformation prior to piercing. A distinct peak occurs at about 0.22 mm, after which the force drops as the needles break through the first layer. Force continues to fall to the next recorded point at 0.39 mm, then behaviour diverges across patches. Some traces rise rapidly as deeper layers compress, while others continue to decrease before rising. The insertion force that achieved a breakthrough was about 1.2–1.5 N based on the dataset accompanying Figure 6. The high sampling rate captured these transitions clearly and supports the identification of the piercing event and subsequent layer engagement.

**Figure 7:**
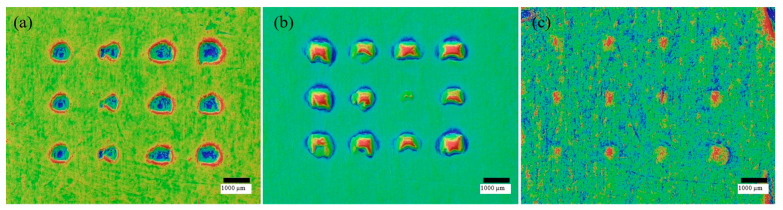
Representative layer images after insertion. (**a**) Patch 1, layer 1 from the 0.15 mm cohort, (**b**) patch 6, layer 2 from the 0.10 mm cohort, and (**c**) patch 7, layer 4 from the 0.20 mm cohort.

**Figure 8:**
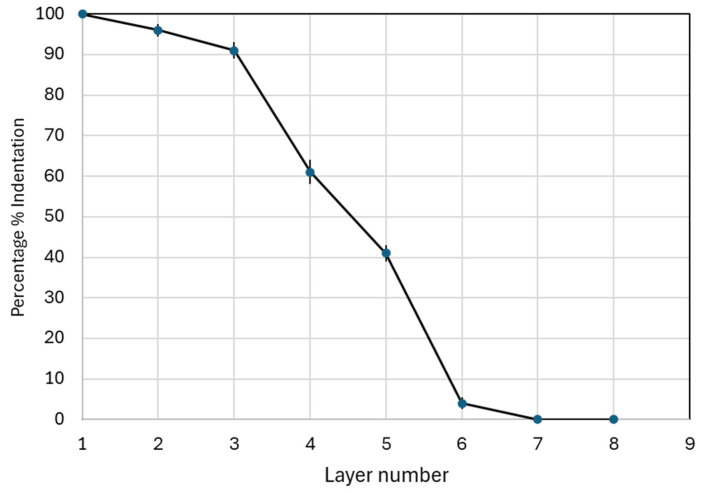
Percentage penetration by layer.

Figure 7 and Figure 8 quantify how far needles propagate through the multilayer stack. The stack comprised eight layers at 127 µm per layer and was unfolded after each test to count perforations. Counts were normalised to the 12-needle array and expressed as a percentage at each depth. The predefined success criterion was 20% penetration. The resulting profile in Figure 8 shows high engagement in the first three layers near 90% on average, followed by a steady decline with depth. Layer 5 corresponds to 635 µm and averages about 40% penetration, which exceeds the success threshold. By layer 6, penetration falls below 5% and no perforations are recorded beyond the sixth layer in the datasets plotted.

Figure 7 provides representative layer images chosen to cover the three wall-thickness cohorts and to illustrate increasing depth. Patch 1 represents the 0.15 mm group and shows a fully perforated top layer. Patch 6 represents the 0.10 mm group and illustrates typical outcomes at layer 2. Patch 7 represents the 0.20 mm group and illustrates a later mid-layer at layer 4. All layers for all patches were imaged and counted, and the full counts underpin the percentages in Figure 8. Figure 8 reports, for each Parafilm layer, the mean percentage penetration across the 15 patches together with the corresponding standard deviation (*n* = 15). The insertion behaviour aligns with the geometry results shown in Figure 5. Bases reproduce close to CAD while tip height undershoots, so the first layers are readily perforated, but deeper engagement is increasingly sensitive to local tip shape and effective height. The force peaks in Figure 6 mark the moment when needles overcome the film surface, and they occur at modest loads for all builds, which supports the practicality of low-force application. Because Parafilm M is recognised as more resistant than skin, the observed profile is a conservative indicator for stratum corneum disruption.

### 3.3. Axial Compression

**Figure 9:**
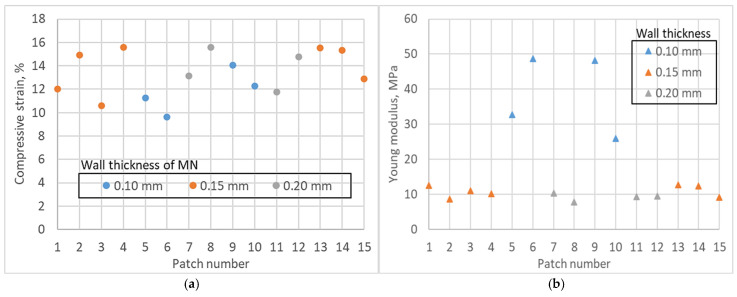
Axial compression outcomes at 150 N. (**a**) Compressive strain per patch of 9.7–15.6% with no fracture. (**b**) Apparent Young’s modulus of 8–48 MPa. Marker types indicate wall thicknesses of 0.10 mm, 0.15 mm, and 0.20 mm.

Figure 9 summarises the array response under axial loading to 150 N. There was no patch fractured at this load. Post-test inspection showed tip shortening and minor bending, but no cracking of the array or base. Compressive strain at maximum load clustered tightly across all builds. Figure 9a shows values between about 9.7% and 15.6% with substantial overlap among wall-thickness groups. The 0.10 mm, 0.15 mm, and 0.20 mm cohorts occupy the same band, indicating similar global compliance of the patch assembly at high load. Any differences are smaller than the visible build-to-build scatter.

The apparent Young’s modulus displayed a wider spread. Figure 9b reports 8–48 MPa across the dataset. The highest moduli occur in the 0.10 mm cohort, including two builds near 48–50 MPa, while most 0.15 mm and 0.20 mm builds cluster around 8–12 MPa. This pattern suggests that thinner walls can stiffen the array in axial compression when geometry and post-cure conditions favour load sharing through the shell rather than local buckling. The dispersion within the 0.10 mm group also shows that stiffness is sensitive to small geometric differences near the tip and to residual curing, consistent with the dimensional variability documented earlier. Combining strain and modulus trends, the arrays tolerate handling scale loads without catastrophic failure and show stiffness that depends on wall thickness more than the peak strain does. These results define a mechanically robust operating window for subsequent use and provide the two quantitative responses.

### 3.4. Factor Effects from the Response-Surface Design

Table 3 compiles the eight measured responses for all 15 builds entered into Minitab. The columns list the percent difference from CAD for the bore base diameter, the mean surface roughness Ra in micrometres, the percent difference from CAD for needle height, the percent difference from CAD for the needle base diameter, the percent height reduction after axial compression, the insertion gradient defined as the slope of percentage penetration versus the Parafilm layer, the compressive strain at maximum load, and the apparent Young’s modulus. Across the dataset, the bore base diameter is mostly oversized with a span of −11.3968% to +16.5541%, which aligns with the optical and profilometry observations in Section 3.1. Mean Ra ranges from 1.77180 to 9.99875 µm, which matches the instrument range reported earlier. Needle height is lower than CAD for every build with a span of −66.3591% to −24.5237%. The needle base diameter varies from −11.3968% to +20.0889%. Height reductions measured after compression have magnitudes from 6.1791% to 32.7013%. Insertion gradients fall between −2.2500 and −1.6548, where more negative values indicate a faster loss of penetration with depth. Compressive strain at maximum load is 9.65% to 15.60% and the corresponding modulus spans 7.80 to 48.79 MPa.

**Figure 10:**
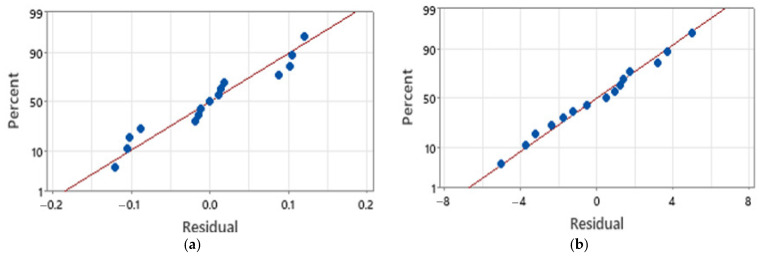
Normal probability plots of residuals. (**a**) Insertion gradient. (**b**) Young’s modulus residuals.

Figure 10 assesses the normality of model residuals for two representative responses. Figure 10a shows the insertion-gradient residuals plotted against the normal distribution. Points lie close to the straight reference line, which supports the normality assumption. The cloud is arranged in three visible bands, which matches the three wall-thickness levels in the design, and the number of points in each band equals the number of runs per level. A slight right-tail departure is present with two influential points. Those points correspond to builds with 0.10 mm walls that showed larger deformation and a steeper loss of penetration with depth. This behaviour is consistent with wall thickness acting as the dominant factor for this response. Figure 10b plots residuals for Young’s modulus. Points track the straight line closely across the full range, which indicates a good approximation to normality for this response. Together, the two outputs illustrate that the response-surface models satisfy the normality assumption, with the only notable deviation confined to the insertion-gradient tail where wall thickness drives influential runs.

**Figure 11:**
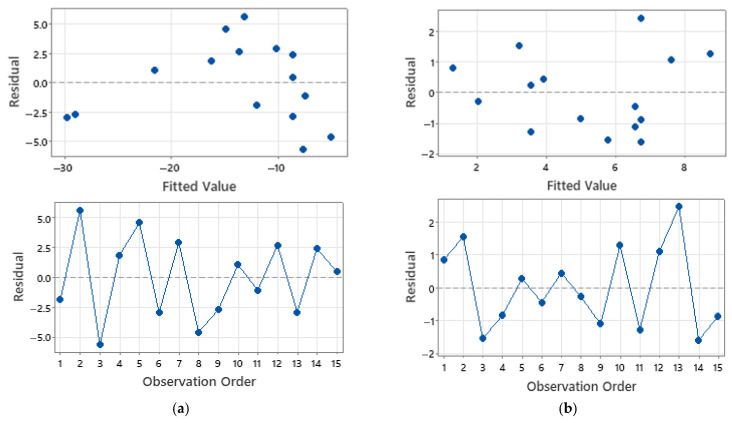
Residual checks for two responses. Top: residuals versus fitted values (**a**) height reduction and (**b**) rough surface. Bottom: residuals versus observation order.

Figure 11 evaluates two model assumptions using residuals for (a) percentage reduction in height and (b) surface roughness. In the residuals versus fitted panels, points fall randomly about zero with no funnelling or curvature, indicating roughly constant variance across the fitted range for both responses. Any visible spread changes are small and not systematic. In the residuals versus order panels, residuals oscillate around zero without trend, step change, or periodic structure across the 15 runs, supporting the independence of errors and the absence of run-order effects. These patterns are consistent with the study-wide statement that residuals versus fitted and versus order were largely pattern-free, and they complement the normality evidence given in Figure 10.

Model fit was strong, with R^2^ ranging from 0.6487 to 0.9733, with most responses above 0.90, which indicates that the models explain most of the observed variation. The significance level was alpha 0.05, and terms were considered significant when the *p* value was less than or equal to 0.05. Five responses met this criterion, listed in Table 4. Wall thickness was significant for all five responses. Wall thickness squared and curing time squared were also significant for specific responses, confirming curvature in factor–response relationships. Within the tested window, curing temperature did not enter as a significant term.

**Figure 12:**
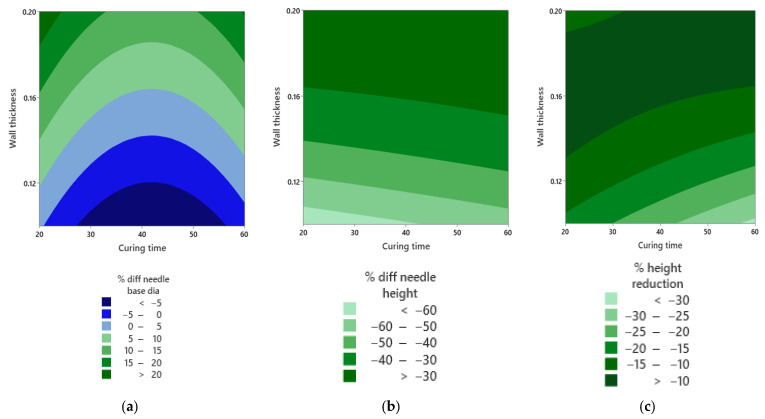
Contour maps of factor effects. (**a**) Base-diameter error. (**b**) Needle-height error. (**c**) Height reduction after compression.

Figure 12a shows that the heat map for the base-diameter error forms two light-blue islands that correspond to the smallest deviations from the CAD target. These islands sit near a wall thickness of about 0.15 mm and at intermediate curing times. Moving toward shorter or longer curing times shifts the response into greener and darker blue bands, which reflects under- or oversizing. The curved shape of these islands is consistent with the significant curing time squared term reported in Table 4. In Figure 12b, the map for needle-height error is predominantly dark green, indicating a smaller magnitude error across much of the space. The darkest region expands as wall thickness moves from thin toward mid-values and as curing time increases, which aligns with the significance of wall thickness and its squared term. This smooth gradation shows that height accuracy improves progressively rather than switching abruptly, again pointing to a curved dependence on thickness within the tested domain. In Figure 12c, the contours for post-compression height reduction show a dark-green band that marks the lowest height loss. This band tracks mid-wall thickness with mid-to-longer curing times. Departing from this band increases the magnitude of height loss, which mirrors the non-linear factor effects seen in the model and supports the role of wall thickness as the primary control variable. The favourable regions in all three maps overlap around 0.15 mm wall thickness with an intermediate curing time. In this window, the arrays achieve an accurate base diameter, improved height fidelity, and reduced height loss after compression.

## 4. Discussion

SLA printing established the performance envelope of the proposed pump-free hollow microneedles before any biological testing, and the fidelity measured signature was apex-limited. Across 15 different runs, cone heights undershot the 1 mm design by −24.5% to −60.5%, whereas base diameters stayed within −11% to +20%, and the bore base was typically +1.2% to +16.6% oversize. This pattern is consistent with a cumulative z-axis error and lateral over-polymerisation at the base. It is also in line with SLA insulin-MN prior research demonstrating that photopolymer arrays can deliver transdermal insulin when geometry is compensated at design and slicing. It is therefore required to introduce lumen-edge negative offsets in the original design, preserve a sharp taper, and tune slicing to protect outlet strategies that map onto prior SLA insulin MN practice [39].

Print physics and build preparation were central to preserving lumen patency and side-port integrity. The print was performed at a 15° tilt angle and 50 µm layers, minimised supports, and kept touchpoints away from flow paths to avoid orifice scarring and tip damage. Profilometry confirmed patent outlets with surface roughness ranging from Ra 1.8 to 10.0 µm without cured rims. The role of orientation as a resolution lever is well documented for SLA microneedles. Angle-induced widening of the effective tip stacking area helps recover tip sharpness even on general-purpose printers, which rationalises the titled building orientation before the experimental matrix [40]. Material choice and post-processing framed the biocompatibility and mechanics. An autoclavable Surgical Guide resin was used and enforced a rigorous wash followed by a post-cure window of 20–60 min at 35–80 °C to stabilise the modulus and minimise extractables. This range mirrors the manufacturer’s evaluation for non-cytotoxic, non-sensitising, non-irritant, and dental device lineage. It also leaves room for device-specific extractables or leachables under intradermal conditions; hence, full post-cure before testing was used.

Beyond the proposed design, micro- and nano-scale 3D printing guidance reinforces the same lever orientation, exposure control, and feature-aware support design as determinants of thin-wall fidelity and outlet preservation. In particular, SLA and µSLA reviews emphasise the trade-off between feature resolution and global extent. It is also a requirement for the need to tune anti-aliasing and cure depth for small radii around outlets and tips, providing an applied broader rationale for the applied process disciplines [41]. With that fabrication foundation, insertion mechanics using Parafilm M^®^ as a conservative layer-resolved surrogate was implemented. The force–displacement curves showed a distinct breakthrough near 0.22 mm at 1.2–1.5 N, followed by force relaxation and depth-dependent divergence. Layer-by-layer counts with eight layers at 127 µm each yielded about 90% perforation across layers 1–3, about 40% at layer 5, which is about 635 µm, and <5% by layer 6. Using the predefined ≥ 20% success criterion, designs met or exceeded success by layers 5–6. This profile demonstrates low-force, near-surface engagement and aligns with Parafilm’s validated role as a rapid, standardisable MN insertion surrogate [30]. Because Parafilm cannot capture dermal backpressure or protein-interference artefacts, it was placed as a screening stage prior to matrices with closer diffusional and hydraulic behaviour. Recent work shows that Strat-M^®^ paired with Parafilm provides reproducible, protein-compatible permeation and insertion readouts that outperform dermatome porcine skin for early screens [31].

Mechanically, the arrays tolerated handling-scale loads. Under 150 N axial compression, no fractures occurred. Compressive strain clustered 9.7–15.6% and apparent modulus in the ranges of 8–48 MPa, indicating a comfortable operational window across wall runs. This is clinically important because ID insulin benefits from earlier absorption and earlier glucose-lowering than subcutaneous routes. Stable mechanics with no buckling or fracture and preserved tip/orifice protect dose reliability and onset timing in routine use [42]. Independent patient studies with purpose-built ID devices corroborate the kinetic advantage and higher early exposure versus subcutaneous delivery, precisely the domain where small geometric drifts can shift placement from viable dermis toward shallow SC. The low force and ≥20% success to about 635 µm therefore map onto clinically meaningful acceleration, not just bench-top penetration [12].

A broader synthesis across insulin MN systems emphasises the same engineering levers we quantified. The proposed lumen oversizing to counter base overshoot and side-port filleting to mitigate local stress and coring follow directly from this literature and explain why we preserved patency after wash/cure while holding the geometry within a manufacturable window [43]. Insertion mechanics analyses also clarify the response-surface outcomes. A surface-response design with 15 runs produced model fits of R^2^ = 0.649–0.973, with wall thickness emerging with a significance of α = 0.05 in five responses, including height error, base-diameter error, post-compression height reduction, insertion gradient, and Young’s modulus. On the other hand, it was found that the curing temperature was not significant within the range. Overlapping favourable regions converged near 0.15 mm of the wall and about 40 min post-cure, balancing height fidelity, insertion robustness, and post-compression retention, exactly the trade-space predicted by slenderness/entry-force theory with a lower tip radius, adequate aspect ratio, and pitch-to-radius of 3.5 [44]. Compression outcome thresholds were found to be dependent on platen geometry, crosshead speed, and the number of needles under load. Adopting individual-needle compression protocols and declaring test conditions alongside penetration/flow metrics will improve comparability and regulatory dialogue as SLA-printed hollow MNs advance [45].

This study was limited to establishing a manufacturable SLA process window and to characterising the geometry, insertion behaviour, and mechanical robustness of the hollow microneedle patch with its self-sealing reservoir. Insulin loading, storage stability, and in vitro release, including release profiles at 37 °C, were not measured. A follow-on quantitative insulin study will instead optimise operational parameters such as insulin formulation and concentration, storage temperature and humidity, residual moisture, packaging and container-closure conditions, reservoir fill volume and number of refill cycles, applied driving pressure and resulting flow rate through the hollow microneedles, patch wear time and application site, and the relationship between these conditions, insulin potency over time, and pharmacokinetic outcomes.

## 5. Conclusions

This study demonstrates a manufacturable route to pump-free hollow SLA microneedle patches with reliable lumen patency and low-force intradermal access. Needle heights undershot the 1 mm CAD target by 24.5–60.5%. Base diameters stayed within −11 to +20%. Bore bases were 1.2 to 16.6% oversize. Tips remained open after wash and cure with profilometry Ra 1.8 to 10.0 µm. Parafilm M showed a breakthrough at 0.22 mm with a peak force of 1.2 to 1.5 N. Penetration reached 90% across layers 1 to 3 at 0.127 mm each. Penetration was 40% at layer 5 at 0.635 mm and under 5% at layer 6 at 0.762 mm. The 20% success criterion was met at clinically relevant depths. The reservoir volume is 117.81 mm^3^. The fill is 0.118 mL and 11.8 insulin units. The patch outer diameter is 15 mm, and the reservoir is 11 mm, which preserves an adhesive margin and applicator fit.

Under 150 N axial compression, no fractures occurred. Compressive strain was 9.7 to 15.6%. The apparent modulus was 8 to 48 MPa. A 15-run Box–Behnken study gave model fits with R^2^ from 0.6487 to 0.9733. Wall thickness was the dominant lever across five responses. Post-cure temperature was not significant within the tested range. Favourable settings converged near 0.15 mm of the wall and 40 min post-cure. These controls reduce height error, preserve patency, and limit post-compression height loss. They also stabilise insertion behaviour at a pitch-to-radius ratio of 3.5 and support repeatable flow through the side port. Progress toward clinical translation now moves beyond Parafilm. Testing on Strat M and dermatome skin with back pressure and flow readouts will refine dosing under tissue-like resistance. Ex vivo and in vivo pharmacokinetic bridging will link insertion and mechanics to onset and exposure. A focused design of experiments on exposure and anti-aliasing will lock lumen patency in the chosen window.

## Figures and Tables

**Table 1:** Summary of design features of MN patches.

Design Element	Rationale/Criteria
Through hole	An internal channel not blocked by resin to allow for efficient insulin flow [37].
Solid cone-shaped base needle tip	A solid cone-shaped tip can withstand the highest compressional forces and provides the easiest insertion [38].
Orifice located on needle shaft	A side-opened orifice greatly reduces clogging of the needle with skin tissue and hence improves drug flow [15].
Outer diameter	15 mm—to be compatible with the uPATCH^TM^ universal MN applicator.
Reservoir	A cavity to store insulin is provided with chamfers at the needle entry for ease of fluid flow. Capacity to store at least 10 units of insulin.
Pitch-to-radius aspect ratio	3.5.
Needle height	Preferably <1500 μm (revise the above discussion).
Size of opening	Small enough to avoid loss of drug in cases of partial insertion.
Base plate	Base plate is larger than the reservoir to secure the patch in place with an adhesive sticker.

**Table 2:** Factors and levels defining the response-surface domain.

Factor	Levels	Units	Notes
Wall thickness	0.10, 0.15, 0.20	mm	Manufacturable and lumen-patent levels. Pitch values tied to these levels to maintain pitch-to-radius of 3.5.
Post-cure time	20, 40, 60	min	Within curing unit capability and material guidance to expose time effects.
Post-cure temperature	35, 60, 80	°C	Within curing unit capability and material guidance to expose temperature effects.

**Table 3:** Measured responses for all 15 builds.

Patch	% Diff Through-hole	Mean Ra (µm)	% Diff Needle Height	% Diff Needle Base	% Height Reduction	Insertion Gradient	Compressive Strain at Fmax (%)	Young’s Modulus (MPa)
1	7.0272	2.15282	−37.7059	20.0889	−13.8386	−2.1905	12.00	12.53
2	16.5541	4.77575	−32.8250	10.0410	−7.5159	−2.0714	14.90	8.71
3	13.6077	4.24242	−28.8122	13.0008	−13.3020	−2.1429	10.60	11.07
4	8.1529	4.15740	−32.4072	7.7731	−14.4208	−2.1429	15.57	10.19
5	1.1254	3.81850	−66.3591	−3.9035	−10.3267	−2.1905	11.24	32.80
6	2.0300	6.12222	−50.4950	0.5727	−32.7013	−2.1071	9.65	48.79
7	8.9478	4.37838	−27.5420	19.5655	−7.2029	−2.2500	13.16	10.47
8	10.5489	1.77180	−25.6737	17.7654	−9.5859	−2.2024	15.60	7.80
9	14.9613	5.47717	−64.5962	−9.7717	−31.6732	−1.7500	14.05	48.20
10	5.0916	9.99875	−62.6381	−11.3968	−20.5217	−1.6548	12.30	26.02
11	12.0193	2.28385	−24.5237	19.1022	−8.4684	−2.2024	11.76	9.35
12	1.5742	8.71082	−28.1032	11.0312	−10.9279	−2.1905	14.77	9.48
13	8.0408	9.17765	−32.5828	−2.2769	−11.4801	−2.1429	15.53	12.85
14	7.7026	5.14190	−28.9758	−0.5369	−6.1791	−2.1667	15.32	12.43
15	6.3473	5.86445	−34.9782	6.6710	−8.1221	−2.1548	12.91	9.12

**Table 4:** Statistically significant terms with practical readings.

Response Variable	Significant Factor Term(s)	*p* Value	Practical Reading
Percent difference in needle height	Wall thickness	0.00	Thickness controls height accuracy.
	Wall thickness squared	0.003	Curved thickness effect points to an interior optimum.
Percent difference in needle base diameter	Wall thickness	0.001	Thickness influences base sizing error.
	Curing time squared	0.012	Mid-curing times minimise error, matching the islands in Figure 12a.
Percent height reduction	Wall thickness	0.014	Mid-thickness reduces post-compression height loss.
Insertion gradient	Wall thickness	0.029	Deeper penetration performance depends on thickness.
Young’s modulus	Wall thickness	0.00	Thin walls show higher axial stiffness.
	Wall thickness squared	0.004	Stiffness varies non-linearly with thickness.

## Data Availability

The datasets are available from the corresponding author on reasonable request.
